# A cross-sectional study of lactation room quality and Dutch working mothers’ satisfaction, perceived ease of, and perceived support for breast milk expression at work

**DOI:** 10.1186/s13006-021-00415-y

**Published:** 2021-09-06

**Authors:** Sjoukje A. van Dellen, Barbara Wisse, Mark P. Mobach, Casper J. Albers, Arie Dijkstra

**Affiliations:** 1 Department of Psychology, University of Groningen, Groningen, The Netherlands; 2 Institute of Future Environments, Hanze University of Applied Sciences, Groningen, The Netherlands; 3 Department of Management & Marketing, Durham University, Durham, UK; 4 Faculty of Management and Organization, The Hague University of Applied Sciences, The Hague, The Netherlands

**Keywords:** Breastfeeding, Breastfeeding duration, Maternal employment, Breast milk expression, Lactation room, Facility management, Netherlands

## Abstract

**Background:**

The challenge of combining professional work and breastfeeding is a key reason why women choose not to breastfeed or to stop breastfeeding early. We posited that having access to a high-quality lactation room at the workplace could influence working mothers’ satisfaction and perceptions related to expressing breast milk at work, which could have important longer term consequences for the duration of breastfeeding. Specifically, we aimed to (1) develop a checklist for assessing the quality of lactation rooms and (2) explore how lactation room quality affects lactating mothers’ satisfaction and perceptions. Drawing on social ecological insights, we hypothesized that the quality of lactation rooms (operationalized as any space used for expressing milk at work) would be positively related to mothers’ satisfaction with the room, perceived ease of, and perceived support for milk expression at work.

**Methods:**

We conducted two studies. In Study 1 we developed a lactation room quality checklist (LRQC) and assessed its reliability twice, using samples of 33 lactation rooms (Study 1a) and 31 lactation rooms (Study 1b). Data were collected in the Northern part of the Netherlands (between December 2016 and April 2017). Study 2 comprised a cross-sectional survey of 511 lactating mothers, working in a variety of Dutch organizations. The mothers were recruited through the Facebook page of a popular Dutch breastfeeding website. They completed online questionnaires containing the LRQC and measures aimed at assessing their satisfaction and perceptions related to milk expression at work (in June and July 2017).

**Results:**

The LRQC was deemed reliable and easy to apply in practice. As predicted, we found that objectively assessed higher-quality lactation rooms were associated with increased levels of satisfaction with the lactation rooms, perceived ease of milk expression at work, and perceived support from supervisors and co-workers for expressing milk in the workplace.

**Conclusions:**

The availability of a high-quality lactation room could influence mothers’ decisions regarding breast milk expression at work and the commencement and/or continuation of breastfeeding. Future studies should explore whether and how lactation room quality affects breastfeeding choices, and which aspects are most important to include in lactation rooms.

**Supplementary Information:**

The online version contains supplementary material available at 10.1186/s13006-021-00415-y.

## Background

Research findings have shown that the challenge of combining working and breastfeeding is one of the main reasons why women do not breastfeed their babies or stop breastfeeding early [1]. To support women continuing to breastfeed their babies when they return to work, organizations may, among other things, offer paid breastfeeding breaks and a lactation room. Maternity protection legislation regarding these aspects can differ per country [2]. According to research by the International Labour Organization, the provision of paid breastfeeding breaks is included in the legislation of 71% of the countries, and the provision of a lactation room is included in the legislation of only 31% of countries worldwide [2].

In the Netherlands, where this research took place, breastfeeding rates are relatively low. In 2018, the percentage of exclusive breastfeeding (operationalized as still receiving breast milk, without receiving infant formula) and any breastfeeding at 6 months of age were only 19 and 28% respectively [3]. At the same time, the number of working women in the childbearing age is high: in 2019, 82.1% of Dutch women in the ages of 25 to 45 were employed [4]. Mandatory paid maternity leave in the Netherlands is 16 weeks, with minimally 10 weeks postnatal leave (Article 3.1, paragraph 1–3 of the *Labour and Care Act*). Subsequently, when a breastfeeding mother returns to work, she is entitled to paid breastfeeding breaks until her infant is 9 months of age; moreover, her employer is responsible for providing a suitable, lockable, and private space for this purpose (Article 4.8, paragraph 1 of the *Working Hours Act*). However, such legislative provisions cannot guarantee that a lactation room will actually be present for the majority of mothers. For example, studies have shown that 32% of Dutch working women who breastfeed do not have any access to a lactation room [5] and 24% of women have access to a lactation room that cannot be locked [6]. Clearly, despite legislation, there appears to be considerable room for improvement regarding the provision and enforcement of suitable breastfeeding facilities worldwide, and in the Netherlands specifically.

To date, scientific studies on the impact of lactation rooms have mainly focused on the effects of the *availability* of lactation rooms, revealing that their mere availability promotes the initiation, duration, and exclusivity of breastfeeding [7]. However, research focusing on the effects of the *quality* of lactation rooms remains a gap in the literature. To encourage further research in this as yet unexplored domain, the current study focussed on two research questions. Firstly, how can a lactation room quality be assessed? Secondly, how is a lactation room quality related to women’s satisfaction and perceptions regarding breast milk expression at work?

### Assessing lactation room quality

A breastfeeding woman needs to breastfeed or express milk regularly during the day to maintain her milk supply and avoid medical problems relating to a build-up of milk. Although, in principle, there could be various ways to achieve this, ranging from taking breastfeeding breaks at home to having the child brought to the workplace. The option that arguably is most prevailing in practice, also in the Netherlands, is for mothers to express breast milk in a lactation room at the workplace. Most official guidelines for designing lactation rooms focus on basic functional aspects, such as hygiene, privacy, and required facilities. For instance, Dutch guidelines specify that a lactation room should be lockable from the inside, hygienic, and offer sufficient privacy. It should be suitable to rest in and should have a (folding) bed or a couch. Moreover, it should offer fresh air and adequate climate control facilities, while not posing specific risks (e.g., the presence of hazardous materials and contaminants) [8]. However, the guidelines only stipulate minimum standards, and the quality of lactation rooms is likely to be influenced by more than just these basic functional aspects. For instance, a recent literature review [9] focusing on office environments concluded that the quality of indoor environments is not only influenced by basic functional aspects, such as indoor air quality, thermal comfort, lighting, acoustics, and office layout, but also by natural, aesthetic, and recreational aspects, i.e., more psychologically oriented aspects. In the case of a lactation room, examples of these aspects are the presence of plants or flowers (natural aspects), paintings or pictures, and coloured walls (aesthetic aspects), books or magazines, and radio or television (recreational aspects). We would argue that such natural, aesthetic, and recreational aspects should also be included when assessing the quality of lactation rooms. In general, natural, aesthetic, and recreational aspects of indoor environments are known to reduce stress [10–13], which impedes the milk ejection reflex [14–16]. Therefore, taking account of natural, aesthetic, and recreational aspects may be important if lactation rooms are expected to facilitate breastfeeding practices. An appropriate method for easily and reliably assessing the quality of lactation rooms does not seem to be readily available at the moment. The development of such a method or checklist was therefore one of the aims of the present study.

### The relationship between lactation room quality and working mothers’ satisfaction and perceptions

A review of the global literature on employer-based programs, policies, and interventions to support breastfeeding among working women was conducted in 2015 [7]. The most common employer-based intervention studied revolved around whether or not organizations provided a private space to express milk. Positive effects associated with access to a lactation space were found for breastfeeding initiation, breastfeeding duration, breastfeeding exclusivity, the use of infant formula, predominant breastfeeding, and job satisfaction. One study [17] found that while access to a lactation space did not have a significant effect on its own, the combination of an available lactation space *and* a refrigerator was associated with continued breastfeeding. This last finding suggests that the mere availability of a lactation room may not be the only important consideration; features or facilities within that room may also be important. In other words, the *quality* of a lactation room may matter for breastfeeding-related outcomes.

We posit that a lactation room could be regarded as an environmental factor that is meaningful in the context of breastfeeding employees. As such, the socio-ecological model, which posits that human behaviour is influenced not only by individual factors but also by socio-environmental factors, provides a relevant theoretical framework [18]. Socio-environmental factors (like individual factors) can influence behaviour directly, but they can also have indirect effects by influencing perceptions, attitudes, or feelings that consequently impact on behaviour [19, 20]. The socio-ecological model has already been successfully applied within multiple breastfeeding studies [21–23]. In the current study we investigated the impact of lactation room quality women’s satisfaction with the room, their perceived ease of milk expression, and their perceived support for milk expression at work from their supervisors and co-workers. Establishing these relationships is important because mothers’ satisfaction and perceptions may influence their breastfeeding behaviour.

First of all, we posit that the quality of lactation rooms may influence women’s overall satisfaction with these rooms. Satisfaction with the physical environment relates to the extent to which the physical environment meets individuals’ physiological, functional, and psychological needs [24, 25]. Accordingly, it can be assumed that the extent to which a lactation room meets the needs of the mothers using it, determines their satisfaction with the room. Because a higher-quality lactation presumably meets the needs of lactating mothers to a larger extent, we expect that lactation room quality is positively related to women’s satisfaction with the room.

Secondly, we posit that the quality of a lactation room may influence mothers’ perceived ease of expressing milk at work. Notably, organizations routinely offer facilities to support the performance of certain activities by employees or the ease of their execution. For example, organizations sometimes offer their employees sport facilities to enable them to engage in physical activities [26–28], or they offer healthy canteen food options to promote healthy eating [29]. Thus, provisions offered by organizations can influence employees’ perceived ease of certain behaviours within specific domains. Similarly, lactation room quality may affect women’s perceptions of the ease with which milk can be expressed at work. Therefore, we expect that lactation room quality is positively related to women’s perceived ease of breast milk expression at work.

Our third and final argument is that the quality of a lactation room may also influence women’s perceptions of support within the workplace for expressing milk. Research in the field of environmental psychology has shown that individuals often derive broader conclusions and judgments about people or organizations from environmental cues [30–33]. The fact that an organization is willing to spend time and money on establishing a lactation room can be viewed as being indicative that an organization deems breastfeeding important and wants to support it. Furthermore, research has shown that environmental cues can communicate not only prevalent types of behaviour in a particular setting, but also what kinds of behaviour are expected [34]. Therefore, the availability of a high-quality lactation room may communicate to employees that managers and co-workers consider women’s milk expression at work to be both customary and desirable. In sum, we expect that lactation room quality is positively related to perceived support of managers and co-workers for milk expression at work.

### Current research

The current research aimed to develop an appropriate method for easily and reliably assessing the quality of lactation rooms (Study 1) and to advance understanding of the effects of lactation room quality on the satisfaction and perceptions of breastfeeding women who work (Study 2). Given the growing number of women joining the labour market and the long-term health benefits of breastfeeding for both mothers and children, acquiring insights into how lactation room design may facilitate the combination of breastfeeding and work seems highly relevant. Study 1 was conducted to develop a checklist for lactation room quality that takes into consideration basic functional aspects of lactation rooms, as well as natural, aesthetic, and recreational aspects. Study 2 aimed to test the hypotheses that lactation room quality would be positively related to women’s satisfaction with the lactation room, their perceived ease of breast milk expression at work, and perceived support of supervisors and co-workers regarding breast milk expression at work. These hypotheses were tested within a large cross-sectional sample of women who expressed milk at work. The current study constitutes an important step in an inquiry of whether and how the quality of lactation rooms may be associated with breastfeeding women’s satisfaction and perceptions regarding breast milk expression at work.

## Methods study 1

### Developing the lactation room quality checklist (LRQC)

We created a concept LRQC for assessing the quality of lactation rooms using a multi-step procedure to ensure the inclusion of the basic functional qualities of these rooms as well as their natural, aesthetic, and recreational qualities. As a first step, we compiled items derived from our review of a variety of online sources and literature providing guidelines and recommendations for the design of lactation rooms. Since there are currently no empirically validated best practices for lactation rooms, we relied on a range of available sources instead. We merged items derived from the Dutch guidelines on lactation rooms [8], a list of best practices in lactation room design produced by the American Institute of Architects [35], and recommendations for lactation rooms formulated by a major breast-pump producer [36]. Items focusing on natural, aesthetic, and recreational aspects were then added to these items to create an initial comprehensive list with items that can be used to evaluate the quality of lactation rooms.

The second step entailed eliciting experts’ inputs. Experts (*N* = 8) were recruited from the network of the first author, and comprised international board-certified lactation consultants and mothers with personal experience of expressing milk at work. Experts received a copy of the list and were instructed to assess whether the wording was clear, whether additional items were needed, and to offer any further feedback to improve the lactation room quality checklist. This process led to two minor adjustments: we added ‘wet wipes’ as an item to the list, and grouped the items into categories to make the LRQC more user-friendly. The third and final step entailed pre-testing the revised LRQC using a think-aloud protocol [37, 38]. Two mothers with personal experience of expressing milk at work pilot-tested the LRQC while talking to the researcher about difficulties or uncertainties with regard to items in the list, which led to some minor changes in wording. The final LRQC contained 35 easily observable items relating to lactation room quality, phrased in a simple and unambiguous way (see Additional file 3 for the full list). We conducted two studies (Study 1a and 1b) to examine the interrater reliability of the lactation room quality checklist.

### Design and procedure study 1a

Thirty-three different lactation rooms in 31 different organizations were rated by three raters to assess the interrater reliability of the lactation room quality checklist. The lactation rooms included in the research were for staff only (no public used lactation rooms were included). Convenience sampling was used to select organizations with lactation rooms. The selected organizations with lactation room facilities mostly included large public sector organizations (22 of the 33 examined rooms were located in such organizations), as small organizations often do not have lactation rooms. The following inclusion criteria were applied in Study 1a: geographical proximity to our location (the northern part of the Netherlands) to enable direct observation of the room, the availability of a lactation room, and the possibility of visiting the organization on one of the three dates that we had selected for the ratings. The principal researcher phoned personnel within the organizations to inquire whether there was an on-site lactation room and to request permission to visit the lactation rooms at these organizations on one of the three selected dates. In total, 72 organizations were approached; 31 organizations agreed to a visit, 30 organizations did not have a lactation room at that moment, 1 organization did have a lactation room, but refused for privacy reasons, and 10 organizations did not know whether they had a lactation room and/or could not identify the correct contact person. Data were collected from December 2016 to January 2017. The three raters, namely the principal researcher (female) and two students (one female and one male) visited all 33 lactation rooms together and filled out a LRQC in each room individually, without discussing their observations with the other raters. In this pilot study the LRQC took on average 3 minutes to complete. The raters were deliberately not provided with training or instructions because the LRQC is intended to be filled in without requiring training or further instruction. If more than one lactation room was present in an organization, all of the lactation rooms were rated, unless the rooms were similarly designed, in which case one room was chosen at random for rating.

### Design and procedure study 1b

**Fig. 1:**
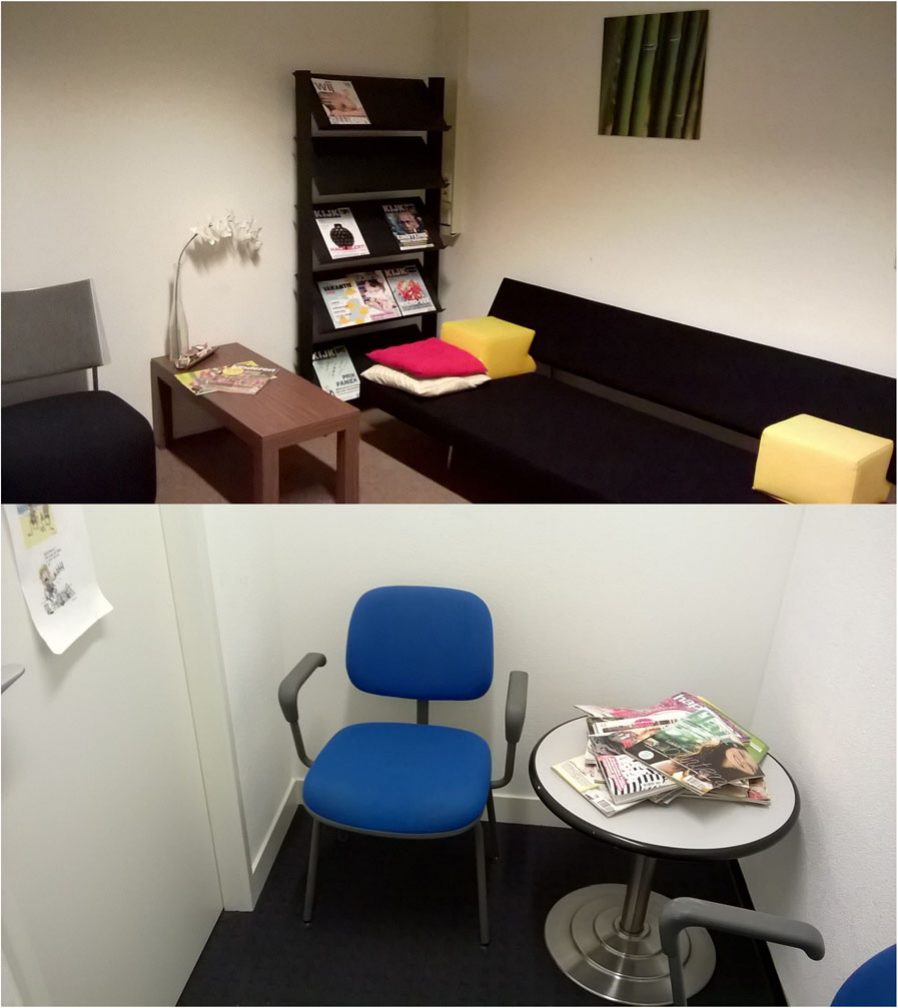
Examples of lactation rooms visited during Studies 1a and 1b

To enable a further examination of interrater reliability of the LRQC, a second reliability study was designed. This study included 31 lactation rooms that were rated by the principal researcher and one mother (a different mother for each lactation room). Of the 31 lactation rooms in Study 1b, 13 rooms had also been examined in Study 1a. The Principal Investigator (PI) visited these rooms again in order to place a recruitment message in the lactation room. In principle, the PI did not fill out the checklist again, unless changes in the lactation room called for it (e.g. one lactation room had had a sink installed since the previous rating). Again, we used convenience sampling to select organizations that were mostly large public sector organizations (21 out of 31 examined rooms were provided by such organizations). The following inclusion criteria were used: geographical proximity to our location (the northern part of the Netherlands) and the availability of a lactation room that was being actively used at the time of the study. The principal researcher phoned personnel within the organizations to ask whether there was a currently used on-site lactation room and to request permission to visit the lactation room. Data were collected from March 2016 to April 2017. The principal researcher visited 41 lactation rooms, filled out a LRQC and left a leaflet in each room, requesting mothers who made use of the lactation room to contact the principal researcher per e-mail to participate in a study about breastfeeding and work. We applied no additional selection criteria (such as frequency of using the lactation room). Mothers who responded were sent an e-mail with a link to an online version of the lactation room quality checklist. No instructions were provided on where the LRQC was to be completed because we assumed that women could fill out the questionnaire without actually being present in the room, given that they expressed milk in these rooms multiple times a day. To prevent participants from guessing while filling out the LRQC, we provided an option for them to indicate that they did not know whether or not the item was present. Participating mothers received a €10 gift certificate. We received responses from mothers for 31 out of the 41 visited lactation rooms, leading to a final sample of 31 lactation rooms that had been observed by the principal researcher and a mother. In this study too, all of the lactation rooms provided by participating organizations were rated unless their designs were similar, in which case, one room was randomly chosen for rating (Fig. 1).

### Data analyses study 1a and 1b

The interrater reliability of the nominal items on the LRQC was assessed with Cohen’s kappa (κ) coefficient using ReCal3, an online tool that computes interrater reliability coefficients for nominal data coded by three or more coders [39, 40]. Responses indicating that the participants did not know whether or not the item was present were considered missing values and were dropped from the analysis (leading to a lower number of ratings for the items concerned, see Table 1).

## Results study 1

Using Altman’s benchmark values [41] for Cohen’s κ, we found that the scores for 24 of the 35 items in the LRQC ranged between moderate and (very) good in both studies (see Table 1). In Study 1a, a poor score (< 0.20) was obtained for one item, fair scores (0.21–0.40) for two items, moderate scores (0.41–0.60) for four items, good scores (0.61–0.80) for nine items, and very good scores (0.80–1.00) for 15 items. The κ values could not be computed for four of the items because of perfect agreement between (two or three) raters. In Study 1b, poor scores (< 0.20) were obtained for seven items, fair scores (0.21–0.40) for two items, moderate scores (0.41–0.60) for five items, good scores (0.61–0.80) for seven items, and very good scores (0.80–1.00) for nine items. The κ values for five items could not be computed because of perfect agreement between two or more raters and the absence of variation in rating scores.

Items that scored below 0.41 (indicating poor or fair reliability scores) in either Study 1a, Study 1b, or in both studies were in principle removed from the LRQC. An exception was made for the five items with a low κ value as a result of a highly skewed distribution (high level of overall agreement among the raters) because these items were also retained [42]. These items, which tended to be always present or always not present in the lactation rooms were ‘a table or surface where you can place a breast pump’, ‘artificial light’, ‘wet wipes’, ‘real plants or flowers’, and ‘ambient lighting’. Ultimately, six items (marked with an asterisk in Table 1) were removed from the lactation room quality checklist. The final LRQC thus comprised 29 items.

**Table 1:** Interrater reliability of the Lactation room quality checklist items (Studies 1a and 1b)

Study	1a			1b		
Description	***n***	% Agreement	Cohen’s Kappa	***n***	% Agreement	Cohen’s Kappa
Access:						
Door with a lock	33	97.97	0.76	31	100	1.00
Lactation room sign	33	95.95	0.91	31	80.60	0.59
Occupied sign	33	83.83	0.65	31	87.10	0.74
Furniture:						
(Folding) bed	33	95.95	0.89	31	96.80	0.93
Couch	33	93.93	0.53	31	100	undefined
Chair	33	100	1.00	31	100	undefined
Table(s) or surface	33	93.93	0.67	31	96.80	0.00
Facilities:						
Coat rack or hook*	33	100	1.00	23	78.30	0.16
Room divider	33	100	undefined	31	100	undefined
Functional socket	33	100	1.00	27	100	undefined
Breast pump	33	95.95	undefined	31	100	1.00
Paper towels	33	91.91	0.83	31	90.30	0.81
Wet wipes	33	97.97	0.76	31	93.50	0.00
Trash can	33	91.91	0.78	30	86.70	0.60
Sink	33	100	1.00	31	96.80	0.92
Fridge	33	100	1.00	31	87.10	0.72
Mirror	33	91.91	0.80	29	100	1.00
Entertainment/relaxation:						
Radio	33	95.95	0.75	31	100	1.00
Television	33	97.97	0.76	31	100	undefined
Books and/or magazines	33	95.95	0.90	31	100	1.00
Facilities to make coffee or tea*	33	97.97	undefined	31	93.50	−0.03
Pillow on the couch or chair*	33	91.91	0.73	30	86.70	0.29
Blanket*	33	81.81	0.25	30	83.30	0.21
Decoration:						
Posters or paintings	33	95.95	0.91	30	86.70	0.72
Artificial plants or flowers	33	97.97	0.89	30	96.70	0.78
Real plants or flowers	33	100	1.00	31	93.50	−0.03
Coloured wall(s)	33	97.97	0.91	30	86.70	0.44
Bulletin board	33	91.91	0.46	31	90.30	0.52
Other decorative items*	33	83.83	0.50	29	79.30	0.13
Windows and lighting:						
Window	33	97.97	0.95	31	100	1.00
Artificial light	33	100	undefined	30	93.30	−0.03
Dimmer	33	100	1.00	30	96.70	0.65
Ambient lighting	33	89.89	0.23	31	96.80	0.65
Climate control:						
Heating	33	72.72	0.51	20	95.00	0.77
Air-conditioning*	33	48.48	0.19	5	80.00	0.55

‘undefined’ means that the κ value could not be computed because of perfect agreement between two or more raters and the absence of variation in rating scores. Removed items are marked with an asterisk (*)

## Conclusion study 1

In Studies 1a and 1b, we developed a practical checklist for quickly, easily, and objectively assessing the quality of lactation rooms. The LRQC’s reliability was tested with two samples, and items considered unreliable were removed. The final list contained 29 items and was found to be reliable regardless of whether a fixed set of raters (Study 1a) or a changing set of raters (Study 1b) filled out the LRQC. After developing the LRQC in Study 1a and 1b, we subsequently used the LRQC to investigate the relationships between lactation room quality and mothers′ satisfaction with the room and perceptions related to expressing milk while at work in Study 2.

## Methods study 2

### Design

We recruited respondents through a call to participate in an online cross-sectional survey posted on the Facebook page of a popular Dutch website that provides breastfeeding information (www.borstvoeding.com). Mothers who used a lactation room at their workplace and had internet access were eligible to participate in the study. We did not specify any criteria that the lactation room had to meet. Therefore, any space used for expressing milk at work, and that was perceived by the users as a lactation room, was regarded as a lactation room in this Study.

### Procedure

Mothers who clicked on the link posted on the Facebook page received further information about the research before proceeding to the survey. The instructions emphasized that participation in the study was voluntary and anonymous and that participants were free to withdraw from the study at any time. Participants were further informed that a total of 15 breastfeeding and/or parenting books would be raffled off and that the winners would be selected from among the respondents who completed the survey. All of the respondents provided their informed consent before continuing to the survey. Data collection occurred in June and July 2017. The study was approved by the Ethical Committee of Psychology (ECP) of the University of Groningen, The Netherlands (reference number: 16394-O).

### Measures

Lactation room quality was measured using the LRQC developed in Study 1. We used the sum score of all items with positive responses (‘yes (present)’; min = 0, max = 29). The internal reliability of the LRQC was acceptable (α = .73) [43]. All items had a positive item total correlation. Exceptions were ‘a door with a lock’ (*r* = .02) and ‘artificial lighting’ (*r* = −.01). These items can be viewed as basic prerequisites for a lactation room. Because Cronbach’s alpha did not change significantly when any of the items were deleted (remaining within a range of 0.71 to 0.74), we decided to retain all items. Mothers were furthermore asked to indicate whether the lactation room was a dedicated lactation or multi-purpose room with the following item: ‘Is this room used only as a lactation room or is it also used for other purposes?’ Answering options were: ‘only as a lactation room’ and ‘also for other purposes’.

Satisfaction with the lactation room was measured with one bipolar item: ‘With the room in general I am. .. (very satisfied - very unsatisfied)’. Perceived ease of breast milk expression at work was measured with one bipolar item: ‘How easy or difficult do you think it is to express breast milk at work? (very easy - very hard)’. Perceived co-worker support was measured with two bipolar items: ‘My co-workers find that I express breast milk at work … (very positive - very negative)’ and ‘My co-workers find that I express breast milk at work … (very important - very unimportant)’ (α = .77). Perceived supervisor support was measured with the same two items, but ‘my co-workers’ was replaced with ‘my supervisor’ (α = .83). All of the items were answered using a 7-point Likert scale, and the responses were recoded so that higher scores reflected more positive outcomes. Finally, demographics (i.e., age, average working hours per week, and education level) were gathered at the end of the questionnaire.

### Data analysis

We performed univariate regression analyses with the quality of lactation rooms as the independent variable and satisfaction, perceived ease of milk expression, and perceived support of co-workers and supervisors as the dependent variables to test our hypotheses. A *p*-value of .05 was considered significant (*p* < 0.0125 after performing a Bonferroni correction for the number of comparisons). The responses of women who did not complete the survey were excluded from the analysis. Responses indicating that the participants did not know whether or not an item of the LRQC was present were considered missing values and were dropped from the analysis. Demographic variables were only used for descriptive purposes.

## Results study 2

### Respondents

A total of 511 mothers completed all of the survey questions. The respondents’ mean age was 32.05 years (SD = 3.98) and they worked an average of 26.59 h per week (SD = 6.53). The majority of mothers reported having a high or medium education level (78.5 and 20%, respectively). A high education level is defined as a Bachelor’s or a Master’s degree, a medium education level as secondary vocational training, secondary general training and pre-university training, and a low education level as everything that is ranked below that. The distribution of women with varying levels of education in the sample, and especially the high proportion of women with high or medium levels of education is not necessarily representative of Dutch women in general. In 2016, 49% of Dutch women between the ages of 30 and 35 years reported having a high level of education and 36% reported having a medium level of education [44]. However, given that breastfeeding is positively related to education levels [45] and that individuals with a higher level of education are more likely to work than those with a lower level of education [46], the relatively high proportion of well-educated women in our sample was not unexpected.

### Quality of lactation rooms

**Fig. 2:**
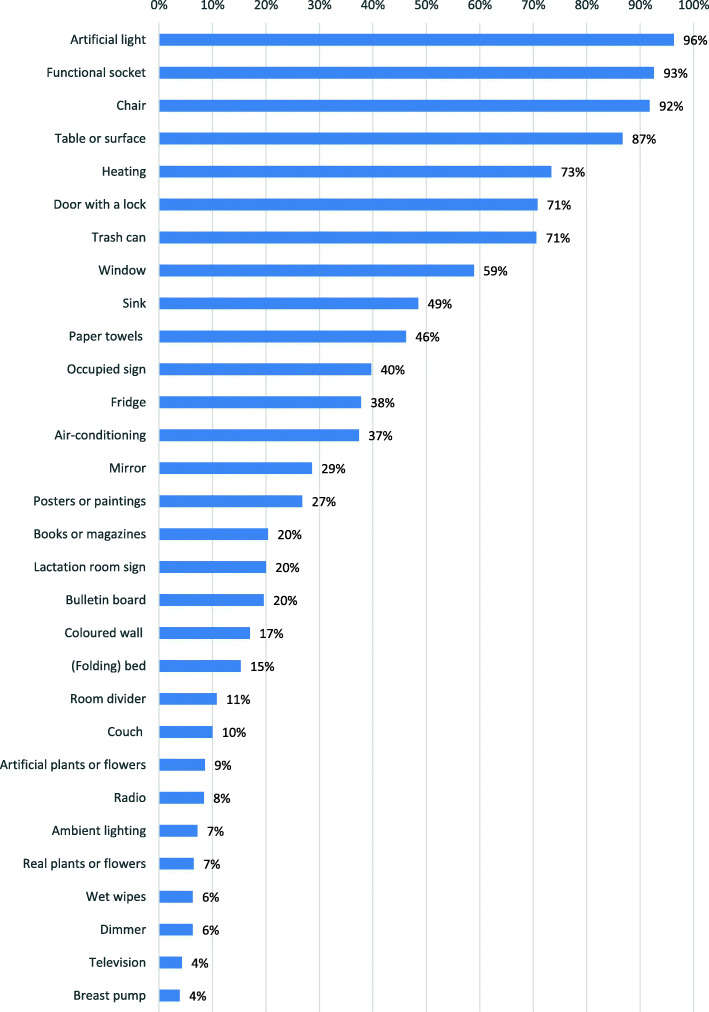
Reported frequencies of lactation room characteristics (*n* = 511)

In our sample only 16.2% of the women used a dedicated lactation room, while 83.8% used a multi-purpose room. Basic functional aspects of lactation room quality were reported to be present in most cases. For example, artificial light, a functional socket, and a chair were present in almost all of the lactation rooms (90% or more). However, natural, aesthetic, and recreational aspects relating to the quality of lactation rooms were less common, see Fig. 2.

Scores for the quality of lactation rooms ranged from 1 to 24 (out of 29 possible positive responses), with a mean score of 10.33 (see Table 2). Notably, dedicated lactation rooms were assigned higher scores relating to their quality than those assigned to non-dedicated rooms (*M* = 12.57 and *M* = 9.90; *F* (1,509) = 39.46, *p* < .001). As we had hypothesized, the correlations between lactation room quality and satisfaction with the lactation room (*r* = .49, *p* < .01), perceived ease of milk expression (*r* = .21, *p* < .01), perceived co-worker support (*r* = .27, *p* < .01), and perceived supervisor support (*r* = .21, *p* < .01) were all significant and positive (see Table 2). Although the correlations between lactation room quality and the dependent variables were significant, they were not particularly strong: most correlations were smaller than .30 (small effect size, according to Cohen’s conventional guidelines [47]), with the exception of the correlation with satisfaction with the room (*r* = .49, medium effect size [47]).

**Table 2:** Means, standard deviations, and correlations of the independent and dependent variables

	Mean	SD	1.	2.	3.	4.	5.
1. Lactation room quality	10.33	3.67	–				
2. Satisfaction	4.68	1.63	.49	–			
3. Perceived ease	4.98	1.52	.21	.36	–		
4. Perceived co-worker support	4.60	1.11	.27	.28	.29	–	
5. Perceived supervisor support	4.55	1.17	.21	.29	.27	.62	–

All of the correlations were significant at the 0.01 level (2-tailed)

### Hypotheses testing

All four hypotheses were supported (see Table 3). Thus, when the quality of lactation rooms was objectively higher, women experienced more overall satisfaction with the lactation room, and they felt that milk expression at work was easier. Moreover, when the quality of lactation rooms was objectively higher, women perceived more support for milk expression from their co-workers and managers at work. Notably, we also performed all of the analyses with age and education level as the covariates. All of the effects remained significant. Therefore, we did not include age and education level as covariates in the final analyses presented in Table 3, because including unnecessary covariates may negatively impact the interpretability of the results [48, 49].

**Table 3:** Univariate associations between lactation room quality and the dependent variables

Dependent variable	***b***	SE B	Beta	***t***	***R***^**2**^	***p***
Satisfaction	.13	.01	.49	12.77	.24	<.001*
Perceived ease	.06	.01	.21	4.85	.04	<.001*
Perceived co-worker support	.07	.01	.27	6.34	.07	<.001*
Perceived supervisor support	.06	.01	.21	4.93	.05	<.001*

df = 1, df error = 509 for all four tests

*Significant at *p* < .0125

## Discussion

The primary goals of this study were (1) to develop a questionnaire for assessing the quality of lactation rooms and (2) to explore the impact of the quality of lactation rooms on breastfeeding mothers’ satisfaction and perceptions related to breast milk expression at work. Study 1 focused on developing the LRQC questionnaire, which was found to be reliable regardless of whether it was filled out by a fixed set of assessors (Study 1a) or a changing set of assessors (Study 1b). Generally, we found that the reliability of the LRQC items was higher in Study 1a than in Study 1b. This could have several causes. First, in Study 1a the raters were all non-users of the room, and therefore more similar than in study 1b, where one rater was the principal researcher, and the other rater was a mother using the lactation room. Second, in Study 1a the LRQC was filled out by all raters at the same time, but in Study 1b the raters filled out the LRQC at different time-points. This means that in Study b, any differences in ratings could have been caused by not only a different perspective (non-user versus user-perspective), but also by actual differences due to changes in the lactation room between the ratings. This could have led to an underestimation of the reliability of the LRQC in Study 1b, and suggests that the reliability estimates found in Study 1b could be considered as conservative estimates. Summarizing, the LRQC was deemed a reliable instrument for quick, easy, and objective assessment of the quality of lactation rooms and thus suitable for application in research and practice. The availability of a reliable measuring instrument is important because it can stimulate and facilitate research on the effects of the quality of lactation rooms on breastfeeding, milk expression at work, and other possible outcome measures. Moreover, it can guide facility managers in organizations in developing evidence-based designs for lactation rooms, and by doing so, improve support for breastfeeding women in their workplaces.

In Study 2, we applied the LRQC within a Dutch sample and investigated the relationship between lactation room quality and women’s satisfaction, their perceived ease of milk expression, and perceived support for milk expression. We found that although most lactation rooms contain at least a chair, a socket, and artificial light, surprisingly several other basic functional aspects, such as a door with a lock, a table, heating, a fridge, or a sink were absent in many of the lactation rooms. Natural, aesthetic, and recreational aspects, such as plants or flowers, paintings or pictures, books or magazines, and radio or television were present only in a minority of the sampled lactation rooms, even though literature suggests that these aspects are important for improving the quality of indoor environments [9]. The low prevalence of dedicated lactation rooms in our sample is also a concern, since we found that on average the quality of a multi-purpose room is significantly lower than that of a dedicated lactation room. All in all, the relatively low quality of the lactation rooms in our study is worrisome and suggests that despite existing regulations [8], the quality of many lactation rooms can be improved. Although only Dutch lactation rooms were examined in this study, there is no reason to assume that the situation would be better in other parts of the world. For example, for a discussion of the gaps between legislation and lactation room quality in the United States, see Dinour and Bai [50]. It is furthermore interesting to note that even though the average quality of the rooms was low (*M* = 10.33 out of a maximum of 29), the mean score for satisfaction was not unfavourable (*M* = 4.68 on a scale from 1 to 7, indicating a score between neutral and a little bit satisfied). This may mean that not all items on the list are equally important or necessary to achieve a certain minimum level of satisfaction. It could also be interpreted as indicative of low expectations with regard to lactation room quality (causing mothers to be relatively satisfied even with a relatively low-quality lactation room).

The results of Study 2 also suggest that the quality of lactation rooms matters because a higher quality is associated with increased satisfaction with the lactation room and mothers’ perceived ease of milk expression at work, suggesting that a high quality lactation room may help mothers to feel more able to express milk at work. Furthermore, the associations between lactation room quality and women’s perceptions of their co-workers’ and supervisors’ support suggests that the quality of lactation rooms can also be interpreted as conveying support for breastfeeding and milk expression at work. In this context, the provision of high-quality facilities may have consequences that extend beyond simply enabling mothers to express milk at work; their availability may signal that milk expression at work is supported by co-workers and supervisors and is considered normative behaviour. Previous studies have shown that the communication of positive norms is especially important for promoting breastfeeding intentions and behaviour. For example, Spitzmueller and colleagues [51] found that perceptions of support at the workplace for breastfeeding and supervisors’ comments about breastfeeding predicted the duration of exclusive breastfeeding. We believe that within organizations, facility management and human resources departments can play an important role in implementing and advancing such policies. Notwithstanding the relevance of other factors, our findings do show that lactation room quality may contribute to removing practical and normative barriers to breast milk expression at work. Organizations should therefore pay attention to the quality of the facilities they offer to their employees who breastfeed.

### Limitations and directions for future research

Although our findings are promising and accord with those in the literature and with our expectations, our study had some limitations. The first entails our use of a cross-sectional design for Study 2. Such designs have two important drawbacks: the influence of common method variance and the inability to draw causal conclusions [52]. With regard to common method variance, we argue that because we used the LRQC to measure the quality of lactation rooms, and scores assigned to items on this list can be assumed to be relatively objective in nature, the associations that we found were most likely not strongly affected by common method variance. Another concern is the directionality of the effects. In the case of lactation room quality, we deem it unlikely that satisfaction, perceived ease of milk expression, or perceived co-workers’ support would have influenced participants’ observations regarding the presence of certain items or options in the lactation room. However, because supervisors are generally able to influence working conditions, it is conceivable that supervisor support in an organization would contribute to a high-quality lactation room. Therefore, reversed causality could be an issue in the relationship between lactation room quality and perceived supervisor support. Moreover, a possible alternative explanation for the associations that we found is that a third factor influenced both the quality of lactation rooms and the outcome variables. For example, a breastfeeding-friendly organizational policy, or a very high personal motivation to breastfeed, could both lead to improvements of the lactation room, *and* impact the outcome variables. Future studies should therefore consider experimental research designs to test the causality of the associations found.

In the current study, we measured only age and education level as biographical variables. Future studies should consider adding more variables that could be helpful in getting insight into the composition of the sample, and in understanding if the effects of lactation room quality on mothers’ satisfaction and perceptions may depend on, for instance, their job and workplace characteristics, the age of the baby, and parity. Future studies could also measure other factors related to instrumental organizational support for breastfeeding (e.g., break time policies, proximity of the lactation room to one’s office, and occupancy rates), as well as factors related to emotional organizational support (e.g., positive norms), to paint a more complete picture of the role that organizations play in facilitating the combination of breastfeeding and work. Finally, future studies could investigate the impact of lactation room quality on a broader range of dependent variables (behavioural outcomes, such as breastfeeding duration and the duration of the period of women’s milk expression at work, would be especially interesting). Moreover, organizationally relevant outcomes could be included. Future studies may examine the effects of lactation room quality on, for instance, perceived organizational support, organizational citizenship behaviours, job satisfaction, exhaustion, and turnover intentions. Elucidating how lactation room quality impacts on organizational outcomes is highly pertinent for organizations dealing with ever-increasing shortages on the labour market due to ageing of society, and could help to create a win-win situation for organizations and their breastfeeding employees.

## Conclusions

We found that lactation room quality is associated with satisfaction and perceptions relating to breast milk expression at work and may thus affect the combination of work and breastfeeding. The ability of increasing numbers of mothers to combine work and breastfeeding successfully offers important societal benefits. Therefore, future studies should explore whether and how lactation room quality affects breastfeeding choices among working mothers, and which aspects are most important to include in a lactation room.

## Supplementary Information

**Additional file 1.** LRQCL rating scores Study 1a. LRQCL rating scores Study 1a

**Additional file 2.** LRQCL rating scores Study 1b. LRQCL rating scores Study 1b

**Additional file 3.** Lactation room quality checklist. Lactation room quality checklist

**Additional file 4.** SPSS Dataset Study 2. SPSS Dataset Study 2

**Additional file 5.** SPSS syntax for Study 2. SPSS syntax for Study 2

## Data Availability

All of the data generated or analysed during this study are included in this published article and in the supplementary information files.

## Abbreviations

ECP: Ethical Committee of Psychology
LQCR: Lactation Room Quality Checklist
TPB: Theory of Planned Behaviour
