# Supplementary material for: A cross-sectional study of lactation room quality and Dutch working mothers’ satisfaction, perceived ease of, and perceived support for breast milk expression at work
Source: Int Breastfeed J. 2021 Sep 6;16:67. doi: 10.1186/s13006-021-00415-y (PMC8422697; doi:10.1186/s13006-021-00415-y)
Supplement: Supplementary file 3 — Additional file 3. Lactation room quality checklist. Lactation room quality checklist [file 13006_2021_415_MOESM3_ESM.docx]

**Additional file 3: Lactation Room Quality Checklist (LRQC)**

| Please indicate what is present in the lactation room: | | | | | | Yes (present) | | No  (not present) |  |
| --- | --- | --- | --- | --- | --- | --- | --- | --- | --- |
| Access: | | | | | |  | |  |  |
| A door with a lock | | | | | | ….. | | ….. |  |
| A sign that indicates this is a lactation room or breastfeeding room | | | | | | ….. | | ….. |  |
| A sign or door hanger that lets you indicate that the room is occupied | | | | | | ….. | | ….. |  |
| Furniture: | | | | | |  | |  |  |
| A (folding) bed | | | | | | ….. | | ….. |  |
| A couch | | | | | | ….. | | ….. |  |
| A chair | | | | | | ….. | | ….. |  |
| A table (s) or surface where you can place a breast pump | | | | | | ….. | | ….. |  |
| Facilities: | | |  |  |  |  | |  |  |
| *A coat rack or hook to hang clothes on* | | | | | | ….. | | ….. |  |
| A room divider | | | | | | ….. | | ….. |  |
| A functional socket | | | | | | ….. | | ….. |  |
| A breast pump provided by the employer | | | | | | ….. | | ….. |  |
| Paper towels / kitchen roll / toilet paper | | | | | | ….. | | ….. |  |
| Wet wipes | | | | | | ….. | | ….. |  |
| A trash can | | | | | | ….. | | ….. |  |
| A sink | | | | | | ….. | | ….. |  |
| A fridge | | | | | | ….. | | ….. |  |
| A mirror | | | | | | ….. | | ….. |  |
| Recreation/relaxation: | | | | |  | |  |  |  |
| A radio | | | | | ….. | | ….. |  |  |
| A television | | | | | ….. | | ….. |  |  |
| Books and/or magazines | | | | | ….. | | ….. |  |  |
| *Facilities to make coffee or tea* | | | | | ….. | | ….. |  |  |
| *A pillow on the couch or chair* | | | | | ….. | | ….. |  |  |
| *A blanket* | | | | | ….. | | ….. |  |  |
| Decoration: | | |  |  |  |  | |  |  |
| Posters or paintings | | | | | ….. | | ….. |  |  |
| Artificial plants or flowers | | | | | ….. | | ….. |  |  |
| Real plants or flowers | | | | | ….. | | ….. |  |  |
| Coloured wall / walls (i.e., not white or beige) | | | | | ….. | | ….. |  |  |
| A bulletin board / magnetic board | | | | | ….. | | ….. |  |  |
| *Other decorative items* | | | | | ….. | | ….. |  |  |
| Windows and lighting: | | |  |  |  |  | |  |  |
| A window | | | | | ….. | | ….. |  |  |
| Artificial light | | | | | ….. | | ….. |  |  |
| Dimmer | | | | | ….. | | ….. |  |  |
| Ambient lighting (for example a twilight lamp) | | | | | ….. | | ….. |  |  |
| Climate control: | | |  |  |  |  | |  |  |
| Heating | | | | | ….. | | ….. |  |  |
| *Air-conditioning* | | | | | ….. | | ….. |  |  |

Note: the items that were removed from the observation list due to insufficient interrater reliability are marked in italics: *A coat rack or hook to hang clothes on, Possibility to make coffee or tea, A pillow on the couch or chair, A blanket, Other decoration, Air-conditioning*.
